# Robotic-assisted ventral hernia repair - a detailed economic evaluation: is it worth it?

**DOI:** 10.1007/s10029-025-03499-1

**Published:** 2025-11-27

**Authors:** Kristian Als Nielsen, Alexandros Valorenzos, Karsten Kaiser, Cathrine Häbel Frandsen, Per Helligsø, Sofie Ronja Petersen, Mark Bremholm Ellebaek, Michael Festersen Nielsen

**Affiliations:** 1 Department of General Surgery, University Hospital of Southern Denmark, Kresten Philipsens Vej 15, Aabenraa, 6200 Denmark; 2 Department of Regional Health Research, University of Southern Denmark, Kresten Philipsens Vej 15, Aabenraa, 6200 Denmark; 3 Department of Clinical Research, University Hospital of Southern Denmark, Kresten Philipsens Vej, Aabenraa, 6200 Denmark; 4 Department of Gynecology and Obstetrics, University Hospital of Southern Denmark, Kresten Philipsens Vej 15, Aabenraa, 6200 Denmark; 5 Department of Clinical Research, Faculty of Health Sciences, University of Southern Denmark, Odense, Denmark; 6 Research Unit of Surgery, Odense University Hospital, Odense, Denmark; 7 Department of Surgery, Horsens General Hospital, Horsens, Denmark

**Keywords:** Robot-assisted surgery, Ventral hernia repair, Cost analysis, Healthcare economics

## Abstract

**Purpose:**

The use of robotic-assisted ventral hernia repair (rVHR) has expanded rapidly, but its economic viability remains debated. This study aimed to provide a transparent cost analysis of rVHR compared with open ventral hernia repair (oVHR), and to identify patient subgroups where rVHR may represent a cost-effective alternative.

**Methods:**

Patients with midline ventral hernias were randomized to rVHR or oVHR. A detailed bottom-up costing approach was applied, including preoperative, intraoperative, hospitalization, and post-hospitalization costs, as well as capital and maintenance costs of the robotic platform. Group-specific mean costs were estimated using log-linear regression models. Cost-consequence and cost-effectiveness analyses were performed, and an interaction model was used to explore cost differences in relation to hernia defect size.

**Results:**

Fifty-six patients were included (29 rVHR, 27 oVHR). Mean total hospital costs were significantly higher for rVHR (€3,539) compared with oVHR (€1,696; cost ratio 2.09, p <0.001). Instrumentation and consumables accounted for the largest share of rVHR costs, while hospitalization represented the largest component in oVHR. Modeling suggested a potential crossover point at a defect size of 56 cm², beyond which rVHR could become relatively more cost-effective. The cost-effectiveness analysis showed that robotic-assisted surgery required an additional €1,149 to reduce hospital stay by one day.

**Conclusion:**

Robotic-assisted ventral hernia repair is associated with substantially higher costs than open repair in our setting. However, rVHR may represent a more cost-effective strategy for larger or more complex hernias, supporting selective use based on patient characteristics and institutional resources.

**Supplementary Information:**

The online version contains supplementary material available at 10.1007/s10029-025-03499-1.

## Introduction

In many Western countries, healthcare spending is increasing at a faster rate than their Gross.

Domestic Product (GDP) [1, 2]. A substantial share of health expenditure is driven by ineffective practices and technologies adopted without robust evidence of benefit, underscoring the need for efficient resource allocation [2, 3]. Ensuring that funds are directed toward interventions that provide the greatest value is essential for maintaining a sustainable healthcare system [1–3].

Since the surgical robotic platform was introduced nearly two 2 decades ago, its financial impact has remained a subject of ongoing debate [4]. In recent years, the use of robotic surgery has increased exponentially, further intensifying discussions about its economic viability and cost-effectiveness. While many studies indicate that robotic minimally invasive surgery (RMIS) is more expensive than laparoscopic or open surgery, some suggest potential cost savings when accounting for factors such as faster recovery, shorter hospital stays, and fewer postoperative complications [5–8]. This discussion is particularly relevant to the repair of large ventral hernias, where the robotic platform, due to its enhanced precision and dexterity, enables procedures that are not feasible with conventional laparoscopy, leaving open surgery as the only viable alternative [9, 10].

In recent studies, robotic ventral hernia repair was shown to be associated with a significantly shorter hospital stay compared to open surgery [11, 12]. While this has clear clinical benefits, it is also particularly relevant from a financial standpoint, as the reduced length of stay may mitigate the high costs associated with the robotic platform, potentially making it more cost-effective than open surgery in some cases.

For publicly funded institutions, the question often remains whether investing in a robotic platform - or purchasing an additional one - is economically justified. However, the existing literature on the cost of robotic surgery in ventral hernia repair is characterized by a lack of transparency, heterogeneity and methodological inconsistencies making it challenging for policymakers and stakeholders to make well-informed economic decisions [13]. To ensure a well-founded rationale for investment, such decisions must be guided by a deliberate, evidence-based assessment.

The objective of this study is to provide a transparent economic evaluation of robotic versus open ventral hernia repair, to identify the patient populations in which robotic surgery may represent the most cost-effective solution, and to support a more evidence-based approach to robotic surgery utilization.

## Materials and methods

The analyses conducted in this study were designed to address the following questions:

1. How do hospital costs compare between robotic-assisted ventral hernia repair (rVHR) and open ventral hernia repair (oVHR)?
2. Is rVHR more cost-effective in specific patient subgroups?

We conducted an economic evaluation using the data from the OVER randomized controlled trial (RCT) comparing open versus robot-assisted ventral hernia repair. The trial was approved by the regional ethics committee (approval number: S-20220106). The study was conducted in accordance with the principles of the Declaration of Helsinki and adheres to the CONSORT guidelines for reporting randomized trials. The trial has been registered at ClinicalTrials.gov (registration number: NCT05906017) on 23 May 2023.

A detailed trial protocol describing the study methodology has been previously published [14]. Participants were stratified based on the preoperative estimated hernia defect size and randomized in a 1:1 allocation ratio to undergo either open or robotic-assisted ventral hernia repair. The study followed an open-label design and operations were conducted between May 2023, and April 2025.

Adults with an ASA classification of 1–3, a clinically and/or radiologically confirmed midline ventral hernia (umbilical, epigastric, or incisional) with an estimated defect diameter of 2–8 cm were included in the study.

Patients were admitted on the day of surgery and discharged based on objective recovery criteria. Follow-up included in-person postoperative assessments at day 1 and 3, as well as 3 month follow up with questionnaire and phone interview.

### Intervention

Surgical techniques varied based on hernia size and surgeon preference within the national hernia guidelines to reflect real-world practice. The open approach included onlay and sublay with retromuscular mesh placement, while the robotic-assisted technique primarily utilized single docking with retromuscular mesh placement, with a few exceptions involving preperitoneal mesh placement. All procedures were performed by four consultant surgeons with extensive experience in both robotic and open surgery. Both techniques were performed under general anesthesia. For selected open cases with large hernias, epidural analgesia and abdominal drainage were employed. Defect size was measured intraoperatively in both the cranio-caudal and transverse directions and used for further analysis. Defect area was calculated using the formula for an ellipse.

### Cost calculation framework

For each individual patient, resource utilization was recorded using standardized templates tailored to the robotic and open surgery groups. Total hospital costs were calculated by summing the preoperative, intraoperative, hospitalization, and post-hospitalization costs, as well as the procedure-specific cost of robotic system usage where applicable.

We used a bottom-up costing approach. Unit prices were identified and multiplied by the estimated quantity of resources used on individual level, based on surgical modality, length of stay (LOS), cut-to-close time, and complication status. Unit costs were obtained from documented sources, including the hospital’s internal accounting and HR salary databases, procurement records and manufacturer price lists, external service contracts (e.g., catering, cleaning, laundry), national drug catalogues (Promedicin.dk), and internal laboratory pricelists. The robotic system cost was derived from the hospital’s purchase and service contract with Intuitive Surgical. A complete overview of all unit costs and their sources is provided in Supplementary Materials S2. Personnel costs were calculated based on basic group-level gross hourly salaries and did not account for individual variations. All cost estimates, except the capital investment for the robotic system, were given in 2024 Danish kroner (DKK) and converted to euro (€) using a fixed exchange rate of 7.45. The robotic system’s capital cost was included in nominal terms based on historical purchase data, as provided by the hospital’s financial office.

#### Preoperative costs

Preoperative expenses included salaries for outpatient clinic staff from both surgery and anesthesiology, as well as diagnostic costs, including e.g. preoperative ECG, and blood testing.

#### Procedure related costs

Operating room expenses included staff salaries for surgeons, anesthesiologists, and nursing personnel, calculated based on the recorded operative time for each individual patient in addition to setup-time and associated administrative tasks. Additional costs comprised instrumentation and consumables, robot-specific drapes, surgical drapes, and expenses related to instrument sterilization and operating room sanitation. Robotic instrumentation costs were calculated by dividing the total purchase price of each reusable instrument by its allowed number of uses, as defined by the manufacturer.

#### Hospitalization costs

Hospitalization costs were calculated based on an informed estimate of daily resource utilization per patient, which was then multiplied by the number of inpatient days. This included staff salaries from the surgical ward, including nurses, doctors, physiotherapists, porters, secretaries, and cleaning and service assistants. Dietary expenses, as well as costs for pharmaceuticals, blood testing, and bed linen, were also included. Nursing salaries were calculated with minute-level precision from admission to discharge, based on average nurse staffing levels in the surgical department. The calculation accounted for wage differences across shifts, including evenings, nights, and weekends.

#### Post-Hospitalization costs

Post-hospitalization expenses included costs related to surgical complications that required hospital intervention, including readmissions and reoperations. The costs were calculated for each individual patient using the same method as described above for the primary operation. Adverse events were defined as readmissions, complications and reoperations.

#### Robotic system costs

We gathered the necessary information from the economics department regarding the initial investment in the robotic system used (Da Vinci Xi), as well as the associated maintenance and service agreements. The expected lifespan of the system was estimated to be 10 years, and annual usage was based on robotic utilization data from the previous year, amounting to 1,300 h of “power on time” across multiple different surgical fields.

*The cost of robotic usage per procedure was calculated as follows:*

$$\:\frac{(\text{C}\text{a}\text{p}\text{i}\text{t}\text{a}\text{l}\:\text{C}\text{o}\text{s}\text{t}\:+\:10\:\text{Y}\text{e}\text{a}\text{r}\:\text{M}\text{a}\text{i}\text{n}\text{t}\text{e}\text{n}\text{a}\text{n}\text{c}\text{e}\:\text{a}\text{n}\text{d}\:\text{S}\text{e}\text{r}\text{v}\text{i}\text{c}\text{e})\text{}}{(10\times\:Annual\:Hours\:of\:Usage)}\times\:Surgery\:Duration$$

### Economic evaluation methods

To assess the economic consequences of robotic-assisted versus open hernia surgery, we employ a combination of Cost-Consequence Analysis (CCA) and Cost-Effectiveness Analysis (CEA).

CCA provides a broad overview of the cost differences between oVHR and rVHR and will be used to present economic outcomes separately.

To quantify cost-effectiveness, a CEA was conducted by calculating the incremental cost-effectiveness ratio (ICER) for length of stay which was the primary outcome of the main RCT.

*The ICER was defined as:*

$$\:ICER=\frac{\varDelta\:Cost\:between\:rVHR\:and\:oVHR}{\varDelta\:\text{E}\text{f}\text{f}\text{e}\text{c}\text{t}\:\left(\text{L}\text{O}\text{S}\right)}$$

This represents the additional cost required to avoid one hospital day through the use of robotic-assisted surgery.

### Statistical analysis

#### Baseline characteristics

Baseline characteristics were compared between the two randomized groups using appropriate statistical tests, selected based on variable type and distribution.

Continuous variables were summarized using either mean and standard deviation (SD) or median and interquartile range (IQR), depending on the distribution. Categorical variables were presented as counts and percentages.

#### Cost consequence analysis

To provide a comprehensive overview of cost data, we report the observed means (crude) to reflect the actual economic burden, and model-estimated means using log-linear regression models with ratios to account for the right-skewed distribution typical of healthcare expenditures. For each cost category, we fitted a linear model with the natural logarithm of the cost as the dependent variable and surgical approach the independent variable. Back-transformed (exponentiated) marginal predictions were used to obtain group-specific mean costs on the original scale, along with 95% confidence intervals and p-values for group comparisons. Model assumptions were assessed graphically using residual plots.

To assess the association between cost and hernia defect size, we fitted a secondary log-linear model that included an interaction term between surgical technique and defect area (Area × Robot). The crossing point - i.e., the defect size at which robot-assisted surgery became more/less costly than open repair - was derived from model coefficients.

All analyses were conducted in R (version 4.3.3, R Foundation for Statistical Computing). A p-value < 0.05 was considered statistically significant.

## Results

### Baseline, operative characteristics and LOS

Sixty patients were randomized for the study, with 4 withdrawing consent, resulting in 56 patients included in the final analysis (29 rVHR, 27 oVHR). No differences were observed between groups regarding baseline characteristics. The median age was 62 years in the rVHR group and 63 years in the oVHR group. BMI was similar across groups (median 31.8 vs. 31.5), as were ASA scores and comorbidity burden based on Charlson Comorbidity Index (Table 1).

**Table 1:** Preoperative patient and hernia characteristics

Variables	rVHR (*n* = 29)	oVHR (*n* = 27)	*p*-value
Patient demographics:			
Age (years), median (IQR)	62 (46,71)	63 (55,69)	0.6^β^
Male sex	19 (66%)	17 (63%)	> 0.9^δ^
BMI (kg/m^2^), median (IQR)	31.8 (4.5)	31.5 (4.7)	0.8^α^
Smoking status			
Never	13 (45%)	9 (33%)	0.7^δ^
Former	6 (21%)	7 (26%)	
Active	10 (34%)	11 (41%)	
Alcohol consumption			
Never	2 (6.9%)	0 (0%)	0.4^φ^
Rarely	6 (21%)	9 (33%)	
1–10 units per week	19 (66%)	17 (63%)	
> 10 units per week	2 (6.9%)	1 (3.7%)	
Comorbidity and risk scores			
ASA grade			
I	3 (10%)	1 (3.7%)	0.6^δ^
II	17 (59%)	16 (59%)	
III	9 (31%)	10 (37%)	
Charlsons co-morbidity index, median (IQR)	2.0 (0.5, 3.5)	2.0 (1.0, 3.0)	0.9^β^
*Comorbidities*			
Hypertension	13 (45%)	12 (44%)	> 0.9^δ^
Heart disease	6 (21%)	3 (11%)	0.5^φ^
Lung disease	8 (28%)	3 (11%)	0.2^φ^
Diabetes mellitus	4 (14%)	4 (15%)	> 0.9^φ^
Dyslipidemia	2 (6.9%)	5 (19%)	0.2^φ^
Previous abdominal surgery	13 (45%)	11 (41%)	0.8^δ^
Hernia characteristics			
Epigastric	4 (14%)	4 (15%)	0.9^φ^
Incisional	5 (17%)	6 (22%)	
Umbilical	20 (69%)	17 (63%)	

Data are reported as median (IQR), mean (SD), or number (%), as appropriate. Between-group comparisons were performed using the independent samples t-test^α^ for normally distributed continuous variables, the Wilcoxon rank sum test^β^ for non-normally distributed continuous variables, and the chi-square^δ^ or Fisher’s exact test^φ^ for categorical variables, as appropriate

Defect size was not significantly different between groups, although the rVHR group had a non-significant median area 2 cm² larger than the oVHR group (Table 2). Mesh size was significantly larger in rVHR (median 225 cm² vs. 150 cm²; *p* = 0.006). Median operative time was also significantly longer for rVHR (129 vs. 80 min; *p* < 0.001). Hernia location, alcohol and smoking habits, and distribution of sex were comparable between groups. The distribution of mesh placement technique differed significantly, with retromuscular positioning being predominant in rVHR (93%) compared to oVHR (59%), and onlay used only in the open group (30%) Table 1 and 2.

The length of hospital stay differed significantly between the groups, with an adjusted mean LOS of 0.46 days for rVHR and 1.96 days for oVHR (*p* < 0.001).

**Table 2:** Intraoperative findings

Variables	rVHR	oVHR	*p*-value
Estimated blood loss (mL)	15.0 (10.0,22.5)	17.5 (2.25,61.25)	0.6^β^
Mesh size (cm^2^)	225 (180, 240)	150 (36, 225)	0.006^β^
Intraoperative hernia defect size (cm^2^)	9 (6, 14)	7 (3, 28)	0.8^β^
Procedure time (min)	129 (115,151)	80 (44,101)	< 0.001^β^
Mesh Placement			
No mesh used	0 (0%)	2 (7.4%)	0.004^φ^
Onlay	0 (0%)	8 (30%)	
Pre peritoneal	2 (6.9%)	1 (3.7%)	
Retro rectus	27 (93%)	16 (59%)	

Data are reported as median (IQR) or number (%), as appropriate. Between-group comparisons were performed using the Wilcoxon rank sum test^β^ for non-normally distributed continuous variables and the Fisher’s exact test^φ^ for categorical variables

**Table 3:** Cost-consequence table comparing rVHR and oVHR

Cost Component	Costs rVHR (95%CI)	Costs oVHR (95%CI)	*p*-value	Cost ratio
Mean total hospital cost	3,539 (3,011 − 4,160) 3,713†	1,696 (1,434-2,005) 1,969†	< 0.001	2.09
Mean total hospital costs without complications	3,356 (2,890–3,895) 3,401†	1,668 (1,430–1,945) 1,936†	< 0.001	2.01
Mean total hospital cost excl. robotic capital	3,209 (2,729–3,774) 3,375†	1,696 (1,434–2,006) 1,969†	< 0.001	1.89
Mean OR personnel cost	883 (804–969) 909†	575 (521–633) 594†	< 0.001	1.54
Mean Hospitalization cost	221 (148–327) 283†	450 (299–678) 924†	0.015	0.49
OR instruments and utensils	1415†	252†	-	5.63
Hourly robot cost (10-yr investment + maintenance)	258†	NA	-	-
Hourly maintenance cost	120†	NA	-	-
Adverse Events*	312†	32†		9.62

The table presents mean hospital costs with 95% confidence intervals, including total costs, costs excluding complications and capital investment, as well as specific cost components such as OR personnel, hospitalization, instruments, and adverse events. All costs are in euros. Crude means marked with (†)

* Readmission, Complications, Reoperations

### Cost outcomes

Robotic-assisted surgery was significantly more expensive across all cost categories except for hospitalization (Table 3). The group-specific mean total hospital cost (including complications, service agreements, and capital investment) was € 3,539 for rVHR and € 1,696 for oVHR (*p* < 0.001), corresponding to a cost ratio of 2.09. When capital costs were excluded, mean costs for rVHR decreased to € 3,209, but still remained significantly higher than oVHR (*p* < 0.001).

Mean hospitalization costs (including nursing salaries) were € 221 for rVHR and € 450 for oVHR (*p* = 0.015). Operation room utensils (including robotic instrumentation) were higher for the rVHR group (€ 1,415 vs. € 252) as were operating room personnel costs (€ 883 vs. € 575; *p* < 0.001).

### Cost-Effectiveness analysis

#### ICER

A cost-effectiveness analysis was performed using LOS as the effect measure. The crude mean total hospital cost per patient was € 3,713 for robotic-assisted surgery and € 1,969 for open surgery, corresponding to a mean difference of € 1,744. Crude mean LOS was 0.48 days for robotic and 2.0 days for open surgery. The resulting ICER was € 1,149 per hospital day avoided, indicating the additional cost required to reduce hospital stay by one day using robotic-assisted surgery.

#### Costs vs. Defect area

Predicted total hospital costs relative to hernia defect area (cm²) are illustrated in Fig. 2. The cost curve for rVHR follows a more linear trajectory, while the curve for oVHR shows exponential growth.

In out setting, the model indicates that when the defect area exceeds approximately 56 cm², the cost curves intersect, and rVHR becomes the more cost-effective option.

Furthermore, we observed that oVHR was initially less costly for small hernias. However, total hospital costs increased more steeply with defect size, by approximately 3% per cm² in the oVHR group (*p* < 0.001), compared to only 1% per cm² in the rVHR group.

## Discussion

This study presents a comprehensive cost analysis and, to our knowledge, constitutes the first full economic evaluation directly comparing robotic-assisted and open ventral hernia repair in an RCT setting. Our findings demonstrate that, although robotic-assisted repair is associated with a shorter hospital stay, it remains significantly more expensive than open repair across all hernia defect sizes in our setting. However, the cost-defect area model indicates that while open repair is initially less expensive, its costs increase at a relatively faster rate with increasing defect size compared to robotic repair. This trend indicates that robotic repair may become relatively more cost-effective in larger hernias. The cost-effectiveness analysis showed that the additional cost required to avoid one overnight hospital stay with robotic-assisted surgery amounted to € 1,149.

Our CCA analysis demonstrates that rVHR is associated with substantially higher total hospital costs compared to open repair, with adjusted mean cost of € 3,539 versus € 1,696 corresponding to a cost ratio of 2.09 (*p* < 0.001). This cost difference remained substantial when excluding the capital investment of the robotic system.

The existing literature on the costs of robotic-assisted ventral hernia repair is highly heterogeneous, often conflicting and largely relies on low-quality evidence with only a few studies based on data from randomized controlled trials. A recent scoping review on costing methodology in rVHR revealed that approximately half of the studies favored the non-robotic comparator in terms of overall costs, while the remaining studies either supported the robotic approach or reported no significant difference [13]. The review also highlighted a lack of transparency in many studies, with selective inclusion of cost components - such as omitting capital investment or service and maintenance costs - thereby limiting comparability and validity. A notable example of a well-executed and transparent cohort study is that by Dauser et al., who found that robotic repair was the less costly option in their setting [15]. However, unlike our study, their patient cohort consisted exclusively of large and complex hernias, and the overall length of stay was substantially longer, which likely contributed to the observed cost-effectiveness of the robotic approach in that context, thereby supporting our interaction model of cost vs. defect area (Fig. 2). Another study by Henriksen et al. also found the robotic approach to be cost-effective in their setting when compared to open incisional hernia repair. Interestingly, however, their analysis excluded key cost components such as the capital investment in the robotic system and expenses related to service agreements [16]. Several other studies have favored the open approach or found no significant difference in terms of overall costs, underscoring the importance of local context and institutional setting in economic evaluations, as cost structures, patient selection, and care pathways can significantly influence outcomes [17–20].

**Fig. 1:**
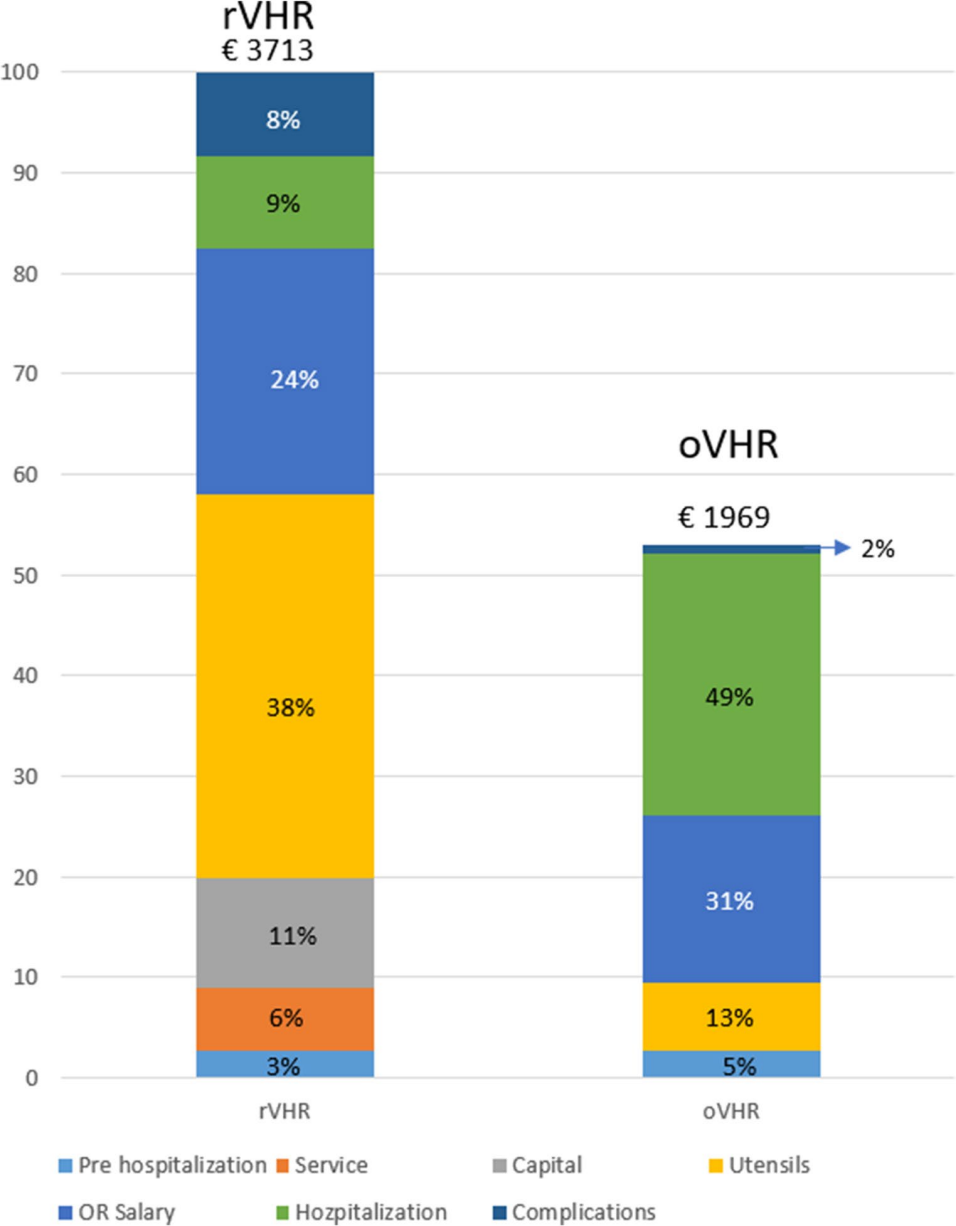
Relative distribution of cost components in rVHR and oVHR. The bars represents the full cost distributed across cost components, including prehospitalization, service agreement, capital investment, utensils, OR salary, hospitalization, and complications. The percentages shown indicate their relative share of the total cost within each modality

Our analyses demonstrate that, in robotic repairs, the largest single cost component was utensils and instrumentation, accounting for 38% of the total mean hospital cost. Service and capital investment contributed an additional 17% combined (Fig. 1). This stands in contrast to the findings of Dauser et al., who identified running costs as the largest cost component - likely due to a lower annual surgical volume in their setting [15].

**Fig. 2:**
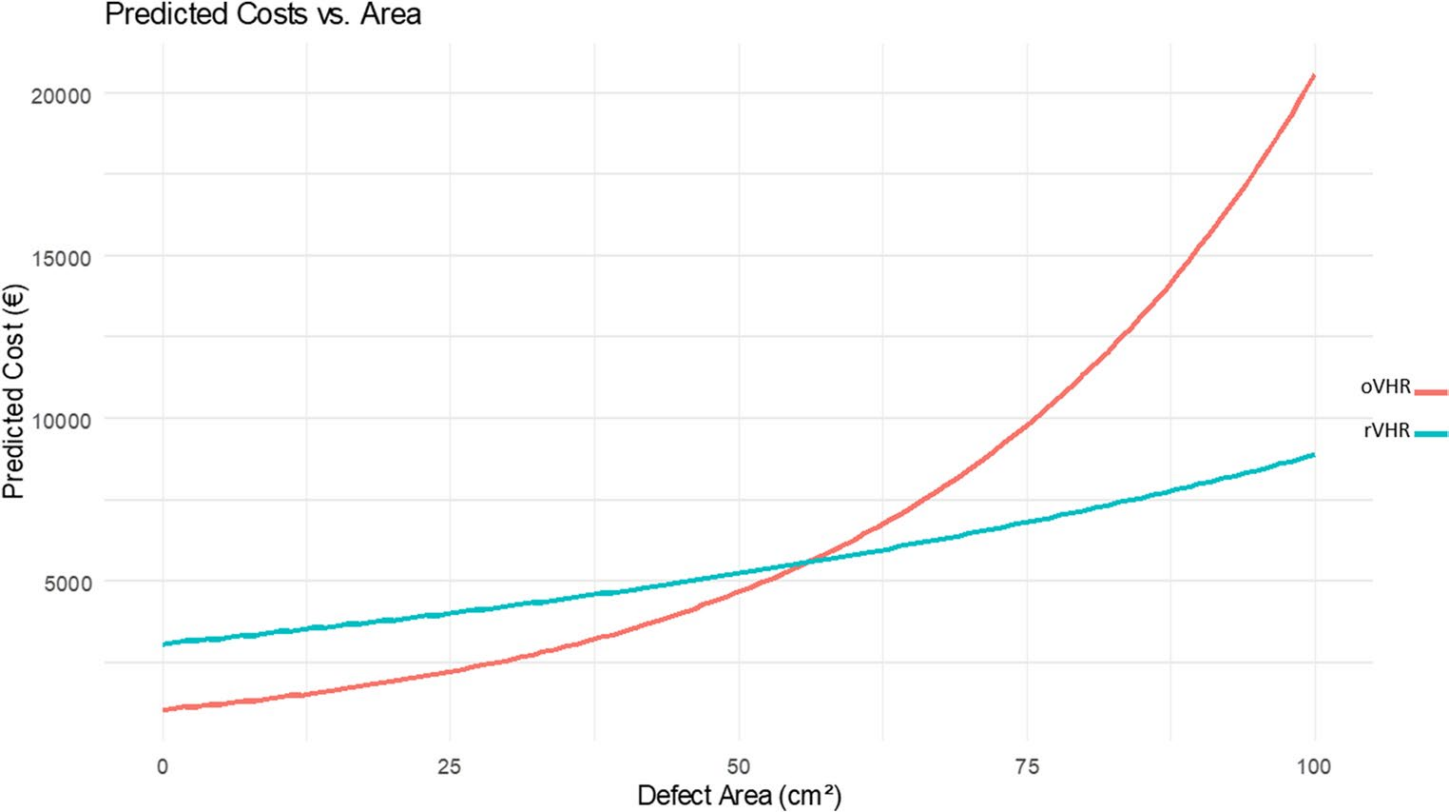
Predicted total hospital costs in relation to hernia defect area for robotic (rVHR) and open (oVHR) ventral hernia repair. The red line represents the cost trajectory for open repair, while the blue line represents robotic repair

Our interaction model (Fig. 2) showed that the cost curves intersected at hernia size around 56 cm², suggesting that robotic-assisted repair may become the more cost-effective option beyond this threshold in our setting, primarily due to the longer length of stay associated with increasing defect size in the open group. However, it is important to note that the largest defect size observed in the oVHR group was only 50.3 cm².

A cost-effectiveness analysis was conducted using LOS as the effect measure. This yielded an incremental cost-effectiveness ratio of € 1,149, indicating the additional cost required to reduce hospital stay by one day when using robotic-assisted surgery compared to open repair.

Interestingly, the costs and cost-effectiveness associated with robotic-assisted surgery are highly dependent on the healthcare setting in which the procedures are performed. In the United States, where healthcare services are generally more expensive than in Europe, the relative cost of using the robotic system may be less pronounced and, in some cases, not significantly higher than alternative approaches [21].

Cost-effectiveness is closely linked to how well the additional capacity generated by the reduced length of stay is utilized. While a shorter hospital stay improves patient satisfaction, it may also free up bed space and enable a higher volume of elective surgeries, potentially resulting in a net economic gain for the institution, while also contributing to shorter waiting lists [22–25].

Furthermore, a high and consistent utilization of the robotic system significantly reduces the relative impact of fixed costs such as capital investment and service agreements, thereby improving the overall cost-efficiency of the robotic platform.

It is also important to acknowledge that many of the advantages of the robotic platform are difficult to quantify in monetary terms. Although our study did not demonstrate significant differences in complication rates or quality of life (to be further explored in a separate forthcoming publication with 6-month follow-up), several other studies have reported that rVHR is associated with fewer complications and reduced postoperative pain compared to open repair oVHR [11, 26, 27]. These potential clinical benefits, while not always easy to capture in cost analyses, should nonetheless be considered when evaluating the value of investing in robotic surgical systems.

As highlighted in a recent scoping review, there are numerous ways to calculate healthcare costs, each with different assumptions and levels of precision [28]. In this evaluation, we employed a bottom-up approach, which involves a detailed and granular component-level estimation of resource use and unit prices. While this method is widely regarded as the most accurate and transparent, it is also resource-intensive and time-consuming and in many cases not feasible. Most other studies on the subject use top down or cost to charge ratios which are aggregate methods that allocates total hospital expenses across different procedures thus provides a broader, less detailed overview from a macro perspective [13, 28].

We estimated the lifespan of the robotic system to be 10 years, consistent with assumptions used in other studies [29]. However, this may introduce some degree of uncertainty, as the system could potentially remain functional for a significantly longer period.

In our setting, the robotic system was donated by a third party. Since this is also the case in other institutions, we have included cost analyses both with and without capital investment. In our specific context, the inclusion or exclusion of capital costs made only a minor difference to the overall cost per procedure.

It is important to note that a variety of financing models exist for robotic systems, each of which can significantly influence the cost per surgery. Common alternatives include leasing agreements and pay-per-use programs that involve little or no upfront investment. In pay-per-use models, the institution typically commits to a minimum number of procedures per year, ensuring a steady utilization of the system [30].

To our knowledge, no published studies compare the cost-effectiveness of different acquisition models for robotic surgical systems. Although it is difficult to determine which model is the most cost-effective overall, we believe that full ownership over the system’s expected lifespan is likely the most economical solution - provided that the system is used at sufficient volume.

### Limitations and future perspectives

Our cost analysis is based on a publicly funded Danish hospital setting, and the generalizability of the findings may be limited by variations in surgical reimbursement models, labor costs, and procurement strategies for robotic systems across healthcare systems. In addition, unit costs were derived from local sources to ensure internal validity, which further strengthens accuracy for our setting but may reduce transferability to other contexts. Nevertheless, the RCT-design, transparency and methodological rigor of our approach provide a robust foundation for comparison and can inform decision-making in other settings, with appropriate consideration of local context.

Pooling of primary ventral hernias (PVH) and incisional hernias (IH) as well as the inclusion of different mesh positions in the open group (30% onlay and 8% no-mesh repairs) may have introduced heterogeneity into our analysis. This heterogeneity could potentially influence operative time, complication rates, and thereby the cost-effectiveness estimates. As highlighted by Stabilini et al. [31], PVH and IH are distinct entities with different patient characteristics, complexity, and long-term recurrence risk, and pooled analysis of these conditions may mask important differences. Similarly, the use of onlay or no-mesh repair in a subset of patients could have reduced operative time and direct costs in the open group compared with retromuscular repairs, potentially biasing against the robotic group.

However, this approach reflects the clinical reality in our center, where surgical decision-making is guided primarily by defect size rather than etiology, in accordance with national Danish guidelines [32]. Both PVH and IH patients are allocated to surgical approach based on defect width, and onlay or sutured repairs remain accepted options for smaller hernias in selected patients. Importantly, randomization ensured that the distribution of PVH versus IH was comparable between groups, minimizing systematic bias. While these factors should be acknowledged as limitations, they also enhance the external validity of our findings by reflecting real-world clinical practice and the actual patterns of resource utilization encountered in daily care.

Our analysis is limited to direct hospital costs related to surgery and inpatient care, and does not account for the additional bed capacity gained through shorter length of stay. Future studies should consider incorporating this aspect, as it may have important implications for hospital efficiency and resource allocation.

Although this is a comprehensive evaluation, the analysis is conducted from a short-term hospital perspective and does not include broader societal costs. A particularly relevant consideration - especially in working-age populations - would have been return to work and productivity loss, which could add a meaningful dimension to the overall cost-effectiveness and provide a more complete assessment.

Moreover, as indicated by the interaction model, our cohort did not include hernia defects large enough to reach the modeled threshold at which robotic-assisted surgery may become the more cost-effective option. Future studies should aim to include patients with larger and more complex hernias to better evaluate the economic advantages of robotic repair in that subgroup.

In addition, future cost-effectiveness studies should also incorporate environmental sustainability metrics, such as waste disposal costs and carbon emissions.

## Conclusion

Robotic-assisted ventral hernia repair was associated with significantly higher total hospital costs compared to open repair, despite a shorter length of stay. While the robotic approach may become relatively more cost-effective in patients with larger hernias, this potential was not fully realized within our setting. For optimal resource allocation, smaller hernias can be effectively treated using the open technique, while robotic-assisted repair should be considered primarily for larger or more complex cases where its advantages may justify the additional cost.

## Supplementary Information

Below is the link to the electronic supplementary material.

Supplementary Material 1

Supplementary Material 2 (XLSX 13.0 KB) 
